# Detection of Feigned Insanity

**Published:** 1857-06

**Authors:** 


*Detection of Feigned Insanity.*—Dr. J. C. Bucknill has some excellent observations on that important forensic point—the diagnosis of insanity. He considers that in the great majority of cases feigned insanity may be detected "by the part being over-acted, in outrageousness and absurdity of conduct, and by the neglect of those changes in the emotions and propensities which form the most important part of real insanity. Sometimes mania is simulated, the man howls, raves, distorts his features and his postures, grovels on the ground, or rushes about his room, and commits numberless acts of violence and destructiveness. If he has had the opportunity of observing a few cases of real insanity, and if he is a good mimic, he may succeed in inducing a person who only watches him for a few minutes to believe that he is in the presence of a case of acute mania; but if the case is watched for a few hours or days, the deception becomes apparent. No muscular endurance, and no tenacity of purpose, will enable a sane man to keep up the resemblance of acute mania; nature soon becomes exhausted, and the would be patient rests, and at length sleeps. The constant agitation, accompanied by symptoms of febrile disturbance, by rapid pulse, foul tongue, dry or harsh or clammy, pallid skin, and the long-continued sleeplessness of acute mania, cannot be successfully imitated. The state of the skin alone will frequently be enough to unmask the pretender. If this is found to be healthy in feeling, and sweating from the exertion of voluntary excitement and effort, it will afford good grounds for suspicion. If after this the patient is found to sleep soundly and composedly, there will be little doubt that the suspicion is correct."

Speaking of chronic mania, Dr. Bucknill observes that it may be imitated; and that if this should be done by an accurate observer of its phenomena, who also happeus to be an excellent mimic, it cannot be denied that he may deceive the most skilful alienist. Fortunately for the credit of psychologists, insanity is rarely feigned, except by ignorant and vulgar persons, who are quite unable to construct and act out a consistent system of disordered mind. It must be remembered that all the features of every case of insanity form a consistent whole, which requires as much intelligence to conceive and imitate as it does to conceive and imitate any dramatic character. The idea which the vulgar have of madness is of quite a different kind. They represent it as a monster, half man, half beast; the emotions they represent unchanged and human; the intelleetual functions they represent entirely perverted, grovelling, and bestial. They think that madness utterly alters the character of a man's perceptions and destroys his judgment, so that he not only ploughs the shore and sows salt for seed, but that he cannot recognise his own son, or avoid the destruction of his life. In more homely cases it will be found that men feigning insanity pretend that they cannot read or write, or count ten correctly, or tell the day of the week, or how many children they have; they answer every question wrongly, which a real lunatic who could be made to understand the question and to answer it at all would certainly answer right. In illustration of these facts, he subjoins the following case of simulated insanity reported by Dr. Snell, in the 'Allgemeine Zeitschrift for Psychiatrie,' December, 1855: "In the House of Correction at Ebernach, a man attempted for some years to escape punishment by imitating insanity. He would not work, he danced round his cell, sang unconnected words and melodies, and made a peculiar booming sound. When any one went into his cell, he put on a forced stupid expression, he glanced at people sideways, but generally fixed his look on the floor or on the wall. To questions he gave either no answers or altogether wrong; for instance, to the question, How many days were there in the week? he answered, Ten. He would not recognize the people whom he constantly saw; he said he had never seen me, and did not know me. When I asked him if he knew who I was, he said a man. I placed before him a keeper with whom he daily bad intercourse, and asked him if he knew who this man was; he said at first he did uot know, and then he said he believed he was a soldier. There could be no doubt there was deception in this case. The unmasked deceiver tried to play his part for some time, and then gave it up.

In the detection of feigned insanity, much stress has been laid by writers upon the suddenness of the attack, as distinguishing it from real insanity, the invasion of which is gradual. This point of diagnosis must be accepted, however, with much caution. Dr. Bucknill has known real cases of mania manifest themselves with the utmost suddenness; he has known patients who went to bed apparently in good health, awake in a state of mania; he has known patients become suddenly maniacal under the influence of exciting and denunciatory preaching, and during other conditions of intense temporary excitement. Doubtless, in all these the brain was previously prepared for the sudden explosion, but the symptoms of latent disease had not been of a nature to attract any observation, and therefore in a diagnostic point of view the sudden outburst of real insanity must be accepted as possible.—*Asylum Journal*, January, 1857.

